# Pulse-Field capillary electrophoresis of repeat-primed PCR amplicons for analysis of large repeats in Spinocerebellar Ataxia Type 10

**DOI:** 10.1371/journal.pone.0228789

**Published:** 2020-03-11

**Authors:** Vera Hashem, Anjana Tiwari, Brittani Bewick, Helio A. G. Teive, Mariana Moscovich, Birgitt Schüele, Khalaf Bushara, Matt Bower, Astrid Rasmussen, Yu-Chih Tsai, Tyson Clark, Karen McFarland, Tetsuo Ashizawa

**Affiliations:** 1 Department of Neurology, Houston Methodist Research Institute, Houston, Texas, United States of America; 2 Movement Disorders Unit, Neurology Service, Department of Internal Medicine, Hospital de Clinicas, Federal University of Paraná, Curitiba, PR, Brazil; 3 Department of Internal Medicine, Federal University of Paraná, Curitiba, Paraná, Brazil; 4 Department of Pathology, Stanford University School of Medicine, Stanford, California, United States of America; 5 Department of Neurology, University of Minnesota Medical Center, Minneapolis, Minnesota, United States of America; 6 Institute of Human Genetics, University of Minnesota Medical Center, Minneapolis, Minnesota, United States of America; 7 Oklahoma Medical Research Foundation, Oklahoma City, OK, USA; 8 Instituto Nacional de Ciencias Medicas y Nutricion Salvador Zubiran, Mexico City, CDMX, Mexico; 9 Pacific Biosciences of California, Inc, Menlo Park, California, United States of America; 10 Department of Neurology and The McKnight Brain Institute, University of Florida, Gainesville, Florida, United States of America; Oklahoma State University, UNITED STATES

## Abstract

Large expansions of microsatellite DNA cause several neurological diseases. In Spinocerebellar ataxia type 10 (SCA10), the repeat interruptions change disease phenotype; an (ATTCC)_n_ or a (ATCCT)_n_/(ATCCC)_n_ interruption within the (ATTCT)_n_ repeat is associated with the robust phenotype of ataxia and epilepsy while mostly pure (ATTCT)_n_ may have reduced penetrance. Large repeat expansions of SCA10, and many other microsatellite expansions, can exceed 10,000 base pairs (bp) in size. Conventional next generation sequencing (NGS) technologies are ineffective in determining internal sequence contents or size of these expanded repeats. Using repeat primed PCR (RP-PCR) in conjunction with a high-sensitivity pulsed-field capillary electrophoresis fragment analyzer (FEMTO-Pulse, Agilent, Santa Clara, CA) (RP-FEMTO hereafter), we successfully determined sequence content of large expansion repeats in genomic DNA of SCA10 patients and transformed yeast artificial chromosomes containing SCA10 repeats. This RP-FEMTO is a simple and economical methodology which could complement emerging NGS for very long sequence reads such as Single Molecule, Real-Time (SMRT) and nanopore sequencing technologies.

## Introduction

Many human diseases are attributed to microsatellite repeat expansions. The mutation of spinocerebellar ataxia type 10 (SCA10) is a complex ATTCT pentanucleotide repeat within intron 9 of the *ATXN10* gene that harbors cryptic repeat insertions [1, 2]. Other large noncoding repeat expansion disorders include Spinocerebellar Ataxia type 8 (SCA8) [3], Spinocerebellar Ataxia type 31 (SCA31) [4], Spinocerebellar Ataxia type 36 (SCA36) [5], Spinocerebellar Ataxia type 37 (SCA37) [6], myotonic dystrophy type 1 (DM1) [7], and myotonic dystrophy type 2 (DM2) [8], fragile X syndrome [9] and fragile X-associated tremor/ataxia syndrome (FXTAS) [10], C9ORF72-associated amyotrophic lateral sclerosis/frontotemporal dementia (ALS/FTD) [11], Friedreich’s ataxia (FA)[12], benign adult familial myoclonic epilepsy (BAMFE) [13], BAFME2 [14), BAFME3 [15], BAFME4 [16], autosomal recessive ataxia related to the cerebellar ataxia neuropathy vestibular areflexia syndrome (AR-CANVAS) [17] and Fuchs endothelial corneal dystrophy (FECD) [18].

Several of these repeat disease sequences have insertions of other repeats, while internal parts of the expanded repeat have not been fully sequenced in others. In the case of SCA10, SCA37, and DM1, the insertion leads to different phenotypes in patients, and the repeat composition may determine the pathogenicity or penetrance of the expanded repeat in SCA8, SCA10, SCA31, SCA37, BAFME, and AR-CANVAS. In SCA10 a normally polymorphic ATTCT repeat, can expand and harbor interruption motifs that can be (ATTCC)_n_, (ATCCC)_n_, or (ATCCT)_n_ [2, 19, 20]. SCA37 is caused by an (ATTTC)_n_ insertion within a polymorphic ATTTT repeat in the non-coding region of DAB1 [6]. While a pure (ATTTT)_7-400_ is non-pathogenic, a complex structure of ((ATTTT)_60-79_(ATTTC)_31-75_(ATTTT)_58-90_) is pathogenic [21]. SCA31 is a complex 2.5 to 3.8-kb pentanucleotide repeat containing components of (TGGAA)_n_, (TAGAA)_n_, and (TAAAA)_n_ insertions within the introns of TK2 and BEAN on chromosome 16 [4]. In DM1 patients, CCG and CGG interruption were identified in the DMPK CTG repeat tract and displayed changes in intergenerational instability and phenotype [22]. SCA8 can harbor Interruptions within the CTG**·**CAG expansion by one or more CCG**·**CGG, CTA**·**TAG, CTC**·**GAG, CCA**·**TGG, or CTT**·**AAG trinucleotides which can duplicate when transmitted from one generation to the next [23]. BAFME is caused by a large (TTTCA) repeat insertion adjacent to the (TAAAA)_n_ repeat in the intronic *SAMD12*, *TNRC6A* and *RAPGEF2* [24]. AR-CANVAS is caused by biallelic pentanucleotide repeat expansion where the normal (AAAAG)_n_ intronic repeat in the gene encoding Replication Factor C1 (RFC1) is replaced by a large (AAGGG)_n_ expansion [17].

Analysis of sequence structure of long repeat tracts larger than 2000 (bp) in SCA10 has been very limited due to the inability to sequence through the repeat using normal sequencing procedures. Some success has been seen using single molecule real time (SMRT) sequencing to determine sequence of SCA10 patient DNA [2] but that technique is limited due to expense, and its intrinsic methodology of single molecule sequencing, which in the case of SCA10 restricts the scope of information to a limited number of expanded repeat DNA molecules, since the normal allele is preferentially sequenced. Nanopore sensor technology also allows for single molecule long sequencing reads (10^4^−10^6^ bases), with minimal amount of sample requirement. However, its effectiveness in reliably sequencing long repeats that possess complex structures has yet to be determined. We demonstrate the use of a combination of repeat-primed PCR in conjunction with the high-resolution pulse field capillary electrophoresis analysis using the FEMTO Pulse Automated Pulsed-Field CE Instrument (Agilent, Santa Clara, CA) to successfully determine sequence content of large expansions of pentanucleotide repeats in SCA10 patients. In addition, this technique was valuable in determining the stable integration of large pentanucleotide expansions into a yeast artificial chromosome in *Saccharomyces cerevisiae*.

## Materials and methods

### DNA samples

DNA samples from blood of 15 SCA10 patients (9 males and 6 females) were obtained with written consent approved by the institutional review boards (IRBs): Houston Methodist IRB, Houston, Texas, USA; University of Florida (UF) IRB, Gainesville, Florida, USA; Ethics Committee of the Parana Federal University, Curitiba, Brazil; IRB at El Camino Hospital, Mountain View, CA; Committee of Clinical Research and Research Ethics at The Instituto Nacional de Ciencias Medicas y Nutricion Salvador Zubiran, CDMX, Mexico. The inclusion criteria were: (1) the genetic diagnosis of SCA10, (2) 18 years of age or older, AND (3) capable of providing informed consent. The exclusion criteria were: (1) having ataxic disorder(s) other than SCA10, OR (2) unwillingness to participate in this study. The diagnosis of SCA10 was established by the referring physician based on clinical phenotype of ataxia and genetic testing. The mean and standard deviation of the age of study subjects were 54 ± 16years (range 34–89), and those of SCA10 repeat expansion size was 1,440 ± 506 repeats (range 822–2360). The Houston Methodist IRB specifically approved this study (Pro00013782).

### DNA isolation

Genomic DNA from SCA10 patient blood was isolated using the DNA Blood and Tissue Kit (Qiagen). Genomic DNA from yeast cultures maintaining a yeast artificial chromosome with SCA10 repeat DNA were isolated using the Yeast DNA Extraction Kit (Thermo Scientific).

### SMRT sequencing

SMRT sequencing was performed as previously described [2]. Briefly, PCR amplicons or unmodified genomic DNA containing SCA10 pentanucleotide repeat were restriction digested and ligated to the SMRTbell adaptors. The SMRTbell fragments of PCR amplicons underwent SMRT sequencing using the PacBio RSII or Sequel IIsequencer. SMRTbell fragments from unmodified genomic DNA were cleaved with CRISPR-Cas9 with gRNA for sequence upstream of the SCA10 repeat. A DNA hairpin adapter with polyA sequence was attached to the Cas9 cleavage site and pulled down using Magbeads carrying polyT DNA oligos [11]. The purified templates containing the SCA10 repeat sequence were subjected to SMRT sequencing.

### FEMTO Pulse Analysis of Repeat-primed PCR (RP-PCR) Products (RP-FEMTO)

RP-PCR was performed as described previously [25, 26]. RP-PCR is an amplification method using fluorescent labelled primers specific to the SCA10 locus upstream of the pentanucleotide repeat paired with pentanucleotide repeat specific primers amplifying at multiple sites within the repeat region. When capillary electrophoresis is performed on the RP-PCR products peaks can be detected where the primers bind indicating region of binding and therefore size and location of the specific repeated DNA.

**Fig 1:**
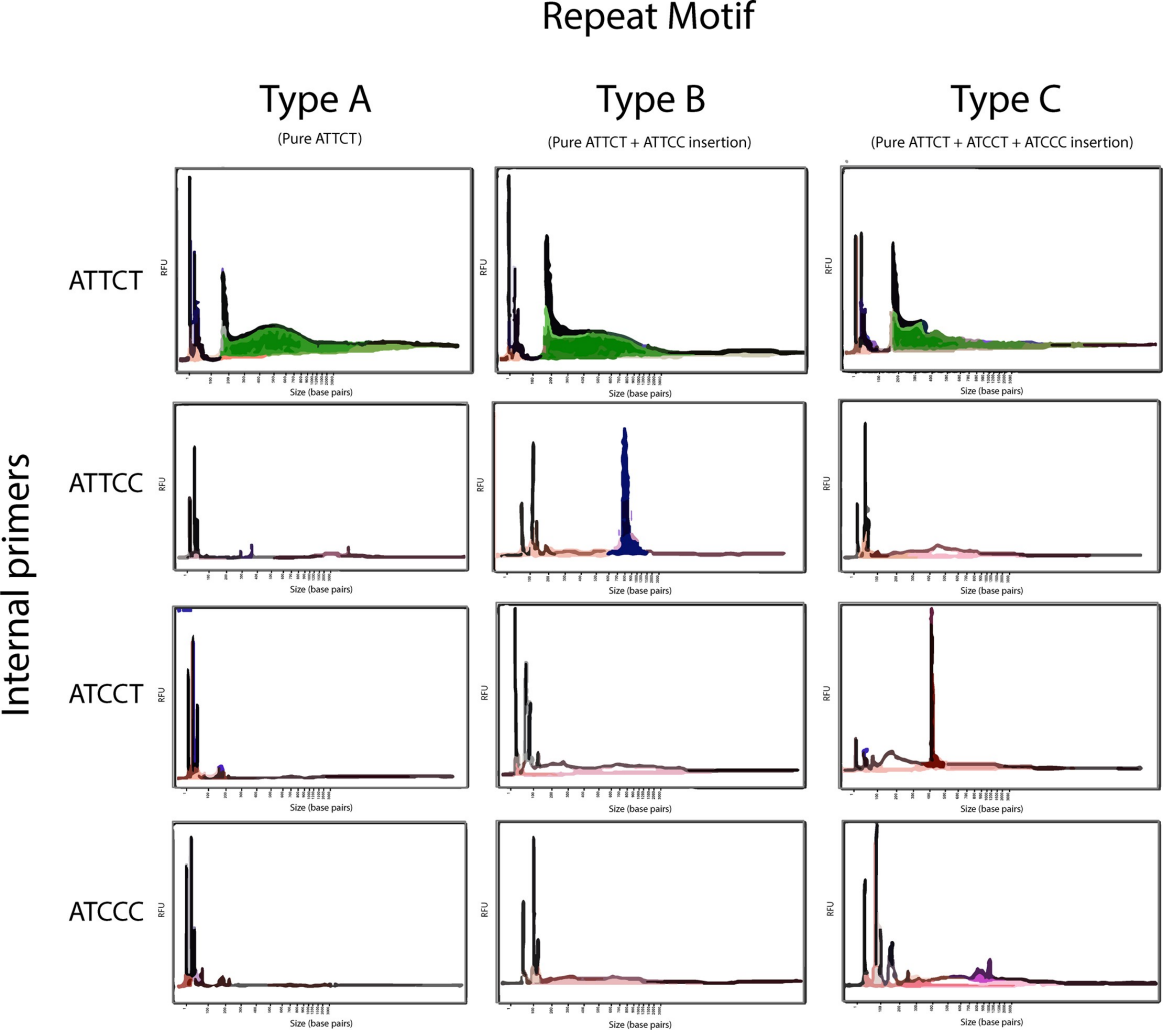
Typical RP-FEMTO results of the various repeat motifs. This figure shows the expected FEMTO-Pulse capillary result using the various internal primers. For the pure ATTCT repeat (Type A repeat), only the ATTCT internal primers results in prominent peaks. ATTCT repeat with an ATTCC insertion (Type B repeat) will result in a prominent peak with the ATTCT internal primer and the ATTCC internal primer. ATTCT with an ATCCT and ATCCC insertion (Type C repeat) will result in peaks with ATTCT, ATCCT and ATCCC internal primers.

Briefly, 100 ng of genomic DNA from blood from SCA10 patients, or 25 ng of yeast genomic DNA containing pentanucleotide repeat expansion on a yeast artificial chromosome (YAC), was added to 1X Amplitaq Gold^™^ 360 Master Mix (Thermofisher), final concentration of 0.6 nM of FAM-tagged forward flanking Primer, 0.6 nM of Tail primer, and 0.06 nM of the respective internal repeat primer in a 30 ul total volume to determine repeat motifs (see Table 1 and Fig 1 for primer and assay details). Internal repeat primers for each known interrupting repeat within the expanded repeats, i.e., (GGAAT)_8_, (AGGAT)_8_ and (GGGAT)_8_, were used to detect (ATTCC)_n_, (ATCCT)_n_ and (ATCCC)_n_ interruptions. The PCR was run in a Veriti ABI PCR Thermal Cycler at the following conditions: Initial Denature 93°C 3’, 17 cycles of 93°C for 15 sec, 61°C for 30 sec and 64°C for 5 minutes, followed by 18 cycles of 93°C for 15 seconds, 61°C for 30 seconds, 64°C for 5 minutes increasing 15 seconds per cycle, followed by a 72°C for 10 minute extension.

**Table 1:** Primers used in RP PCR to detect the various repeat interruptions.

	Primers	Primer Sequence	Purpose
RP PCR of pure (ATTCT)_n_	Forward Flanking primer Tail primer ATTCT primer	5’-FAM-GAAGACAAATAGAAAACAGATGGCAGA-3’ 5’-TACGCATCCCAGTTTGAGACGG-3’ 5’- TACGCATCCCAGTTTGAGACGG(AATAG)8−3’	Detects length of ATTCT repeat
RP PCR of (ATTCT)n with (ATTCC)_n_ interruptions	Forward Flanking primer Tail primer ATTCT primer	5’-FAM-GAAGACAAATAGAAAACAGATGGCAGA-3’ 5’-TACGCATCCCAGTTTGAGACGG-3’ 5’- TACGCATCCCAGTTTGAGACGG(AATAG)8−3’	Detects length of ATTCT repeat
	Forward Flanking primer Tail primer ATTCC primer	5’-FAM-GAAGACAAATAGAAAACAGATGGCAGA-3’ 5’-TACGCATCCCAGTTTGAGACGG-3’ 5’-TACGCATCCCAGTTTGAGACGG(GGAAT)5−3’	Detects length of ATTCC repeat and position within the ATTCT repeat
RP PCR of (ATTCT)_n_ with (ATCCT)_n_ and (ATCCC)_n_ interruptions	Forward Flanking primer Tail primer ATTCT primer	5’-FAM-GAAGACAAATAGAAAACAGATGGCAGA-3’ 5’-TACGCATCCCAGTTTGAGACGG-3’ 5’- TACGCATCCCAGTTTGAGACGG(AATAG)8−3’	Detects length of ATTCT repeat
	Forward Flanking primer Tail primer ATCCT primer	5’-FAM-GAAGACAAATAGAAAACAGATGGCAGA-3’ 5’-TACGCATCCCAGTTTGAGACGG-3’ 5’- TACGCATCCCAGTTTGAGACGG(AGGAT)6−3’	Detects approximate length of ATCCT interruption and position within the ATTCT repeat
	Forward Flanking primer Tail primer ATCCC primer	5’-FAM-GAAGACAAATAGAAAACAGATGGCAGA-3’ 5’-TACGCATCCCAGTTTGAGACGG-3’ 5’- TACGCATCCCAGTTTGAGACGG(GGGAT)3GG-3’	Detects approximate length of ATCCC interruption and position within the ATTCT repeat

RP-PCR product was diluted 1:400 in Tris-EDTA and 2μl was used to run on the FEMTO Pulse analyzer (Agilent, Santa Clara, CA) using both the Large DNA Separation FP-5001 Gel or the small DNA separation gel FP-5201 Gel (Agilent) following the manufacturer’s instructions.

### Analysis

Raw data obtained from running the samples in the FEMTO Pulse System was analyzed using the PROSize Data Analysis Software from Agilent.

## Results

### Comparison of RP-FEMTO data with SMRT sequencing from genomic DNA of SCA10 patients

SCA10 pentanucleotide repeat expansions with (ATTCC)_n_, (ATCCT)_n_, and (ATCCC)_n_ interruptions were observed in SCA10 patient’s DNA [2, 27]. Sequencing of the large repeats by conventional methodology including the NGS did not work due to the large repeat sequence structure. The repeat units within internal sequences of expanded SCA10 repeats were initially identified by long PCR amplification of relatively small SCA10 repeat expansion alleles, shearing of the amplicon, shotgun cloning of sheared fragments, and Sanger sequencing of cloned sequences, which produced histograms of repeat units within the SCA10 expansion [2]. The SMRT sequencing of the same long amplicons provided long single molecule reads of these expanded allele sequences. Comparisons of these two sets of data showed general agreements of the repeat unit composition [2]. Furthermore, in long sequence reads of SMRT sequencing, the basic composition and arrangement of heterogeneous repeat units were maintained between the circular consensus sequences (CCSs) obtained from the genomic DNA of same individuals (unpublished data). Thus, we considered results of the SMRT sequencing of expanded SCA10 repeat as reliable reference sequences. We utilized the RP-FEMTO to identify the various interruptions and determine approximate repeat length of SCA10 pentanucleotide repeat expansion.

**Fig 2:**
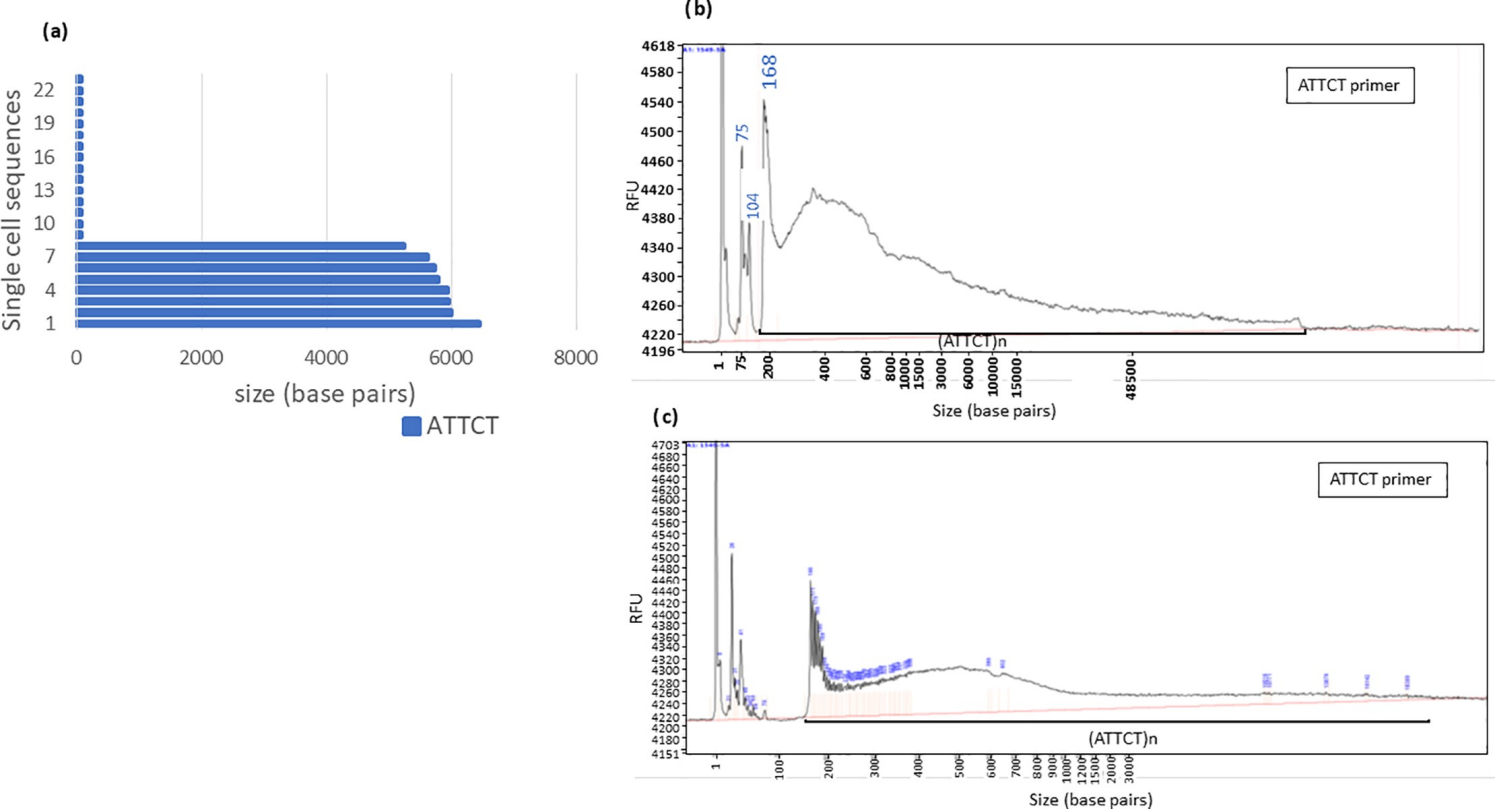
Comparison of SMRT sequencing data with RP-FEMTO results of an (ATTCT)_n_ pentanucleotide repeat expansion from an SCA10 patient. **(a)** SMRT sequencing results for a pure (ATTCT)_n_ repeat from SCA10 patient blood DNA. Single cell sequences, represented by the blue bars, resulted in eight expanded alleles ranging between 6200–6450 bp, and 154 normal length alleles ranging between 60–70 bp (for graph clarity, only 15 normal sequences are shown). **(b)** RP-FEMTO results of same DNA using ATTCT internal primer and running on the large separation gel, capable of detecting fragments up to 165kb. The repeat starts at 170 bp. The main capillary peak is between 6000–8000 bp but can continue to above 15,000 indicating PCR amplification of larger repeats. Repeat length mosaicism was observed with SMRT sequencing data in blood genomic DNA from SCA10 patient. **(c)** RP-FEMTO results of same DNA using ATTCT internal primer and running on the ultrasensitive NGS small separation gel which provides improved sensitivity showing peaks of individual repeat units but is limited to smaller fragments of 100–6000 bp. Considering that the repeat length of this genomic sample was above 7000, this may not provide accurate sizing.

**Fig 3:**
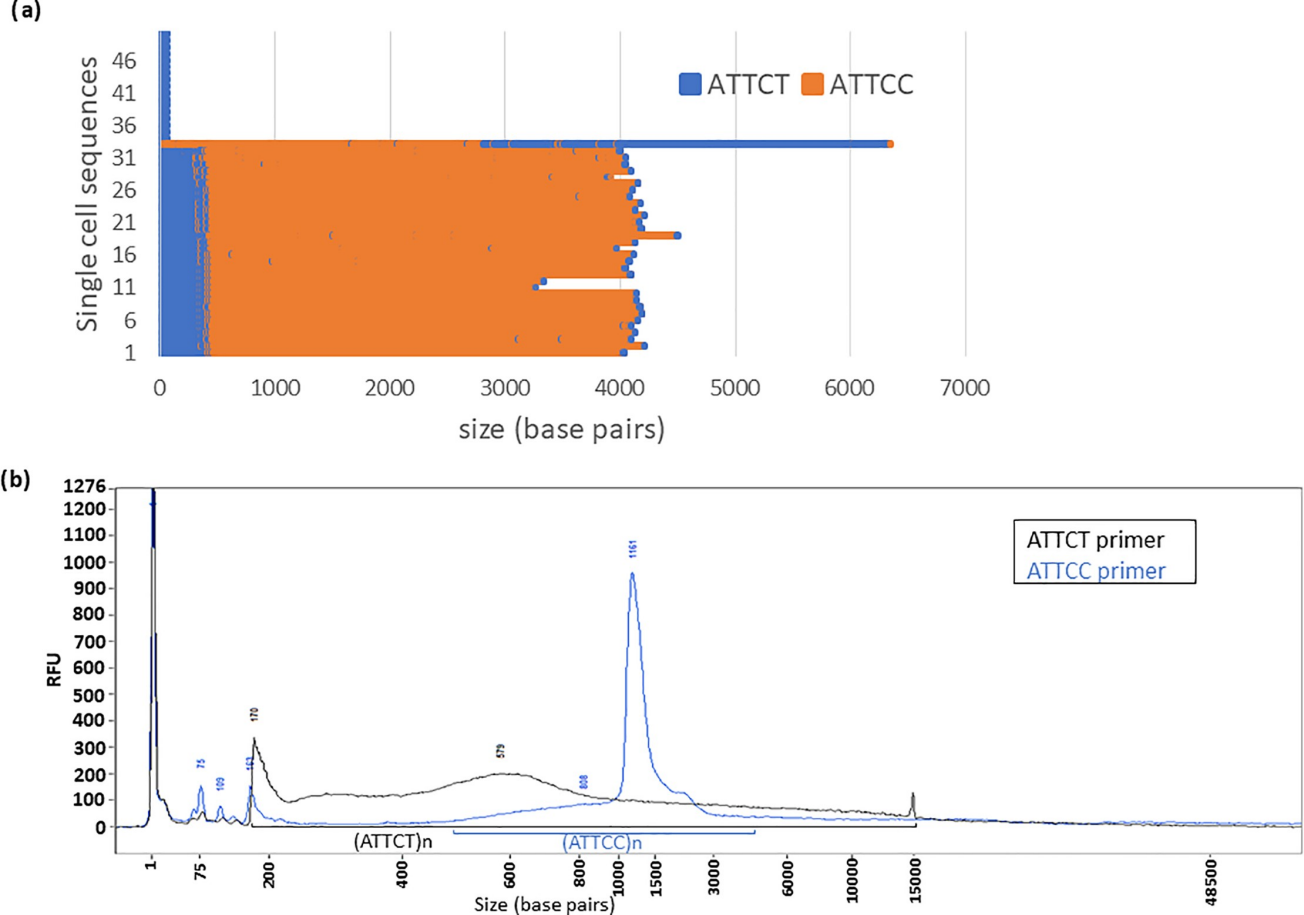
Comparison of SMRT sequencing data with RP-FEMTO results of a SCA10 patient with an (ATTCT)_n_ expansion maintaining an (ATTCC)_n_ interruption. **(a)** SMRT sequencing results for a (ATTCT)_n_ repeat containing (ATTCC)_n_ interruptions from SCA10 patient blood DNA. Single cell sequence results from SMRT sequencing, represented by the blue bars for ATTCT repeats and orange bars for ATTCC repeats, resulted in 33 expanded alleles ranging between 3200–6400 bp (640–1280 repeats), and 174 normal length alleles having 70 bp (14 ATTCT repeats) although only 17 are shown in this figure for graph clarity. **(b)** RP-FEMTO results of same genomic DNA using ATTCT (black line) or ATTCC (blue line) internal primer and running on the large separation gel. Some peaks overlap due to repeat length variation in the blood, which is verified with the SMRT sequencing results. The repeat starts at 170 bp. The total repeat length is under 15000 bp with an insertion of ATTCC repeats of about 3500 bp.

RP-PCR was used to amplify pentanucleotide expansions of SCA10 patients’ DNAs (see Table 1, Fig 1 and method sections). RP-PCR amplicons underwent fragment analysis using the FEMTO Pulse system (Agilent, Santa Clara, CA) which can generate data for fragments up to 165 kb. We used the Large DNA Separation 5001 Gel to detect large expansions over 6000 bp and the Small Separation FP-5201 Gel to detect smaller expansion under 6000 bp (Agilent, Santa Clara, CA). Fig 2 and Fig 3 show comparison of results obtained with the SMRT sequencing and those from the RP-FEMTO analysis.

DNA samples from 12 patients with SCA10 were analyzed by SMRT sequencing. From each patient’s blood genomic DNA, 79 to 269 SMRT CCSs were obtained. We expected that SMRT sequence reads of normal and expanded SCA10 repeat will show the 1:1 ratio. However, expanded alleles were grossly underrepresented: only 2 to 33 of these sequence reads consisted of repeat expansions while the rest of sequence data were of the normal-length alleles. These expanded SCA10 repeats consisted of either (ATTCT)_n_ or (ATTCT)_n_-(ATTCC)_n_-(ATTCT)_1-10_ configurations. With the RP-FEMTO the size of expanded alleles was larger than that detected by SMRT sequencing. Although the RP-PCR may also have an amplification bias toward shorter alleles, the high sensitivity of the FEMTO-Pulse system was able to detect a small number of larger expanded repeats.

For example, SMRT sequencing showed variable lengths of eight molecules consisting of a pure expanded (ATTCT)_n_ repeat ranging from 5400 bp to 6500 bp (Fig 2A). Whereas, SMRT sequencing gives results for limited individual repeat sequences, the RP-FEMTO gives a representation of the conglomerate of a greater number of molecules where the main capillary peak is around 6000 to 8000 bp, but there were also some PCR products more than 10,000 bp long, suggesting that much larger expansions exist in the patient’s DNA but cannot be detected by SMRT sequencing. RP-FEMTO of blood DNA of normal individuals shows a single prominent peak corresponding to 60–70 bp (12–14 pentanucleotide repeats) and no extension of the peak indicating that the RP PCR peak extension observed in the capillary figures of the expanded repeats is specific to the expansion (Data not shown). Southern Data for this sample shows a band of 7000 bp. In a heterogenous population, Southern data would also have a bias towards the repeat lengths that are more prevalent in the genomic population. The RP-FEMTO data and the SMRT sequencing data for SCA10 samples containing (ATTCC)_n_ interruptions show similar results (Fig 3A).

**Fig 4:**
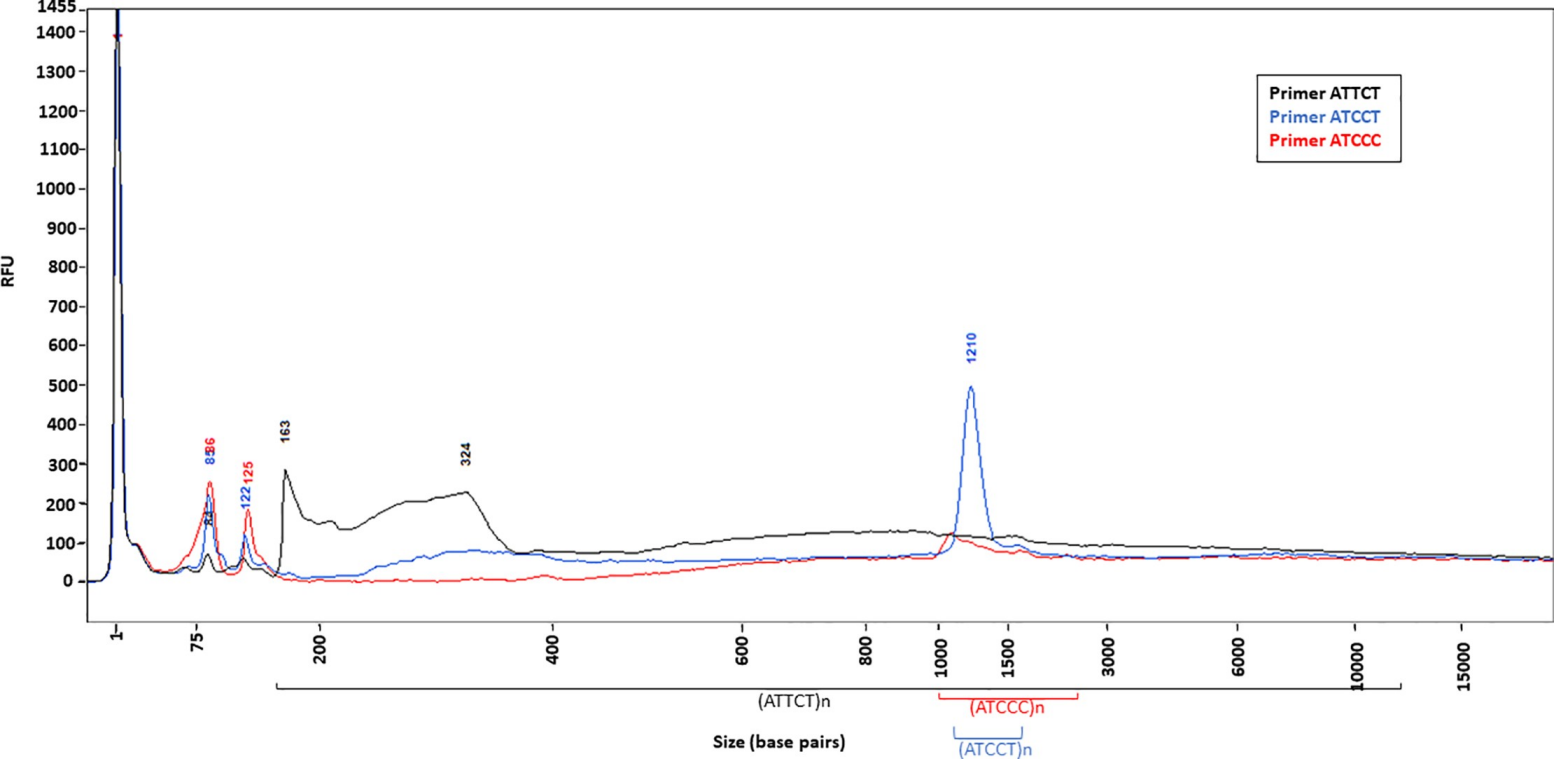
RP-FEMTO results of a SCA10 patient with an (ATTCT)_n_ expansion maintaining an (ATCCT)_n_(ATCCC)_n_ interruption. RP-FEMTO results of genomic DNA using ATTCT (black line), ATCCT (blue line) or ATCCC (red line) internal primer and running on the large separation gel. Some peaks overlap due to repeat length variation in the blood. Repeat starts at 163 bp. RP-FEMTO shows a genomic DNA population containing (ATTCT)_200_ indicated by the black line, followed by (ATCCT)/(ATCCC)_500_ indicated by the blue and red lines.

SMRT sequencing did not yield CCSs of other SCA10 patients’ expanded alleles but showed subreads that did not complete multiple circular sequencing. These subreads identified expansions in the configuration of (ATTCT)_n_-(ATCCT)_n_-(ATCCC)_n_-(ATTCT)_1-10_. Using the RP-FEMTO, we were able to determine a sequence motif of around 1000 bp of (ATTCT)_n_ repeats followed by about 500 bp of (ATCCT)_n_ and (ATCCC)_n_ repeats (Fig 4). In fact, when considering clinical diagnostics, it may be beneficial to do both SMRT sequencing and RP in parallel.

### The use of RP-FEMTO in the determination of stability of repeat length and composition after transformation of large pentanucleotide repeats into a yeast artificial chromosome in *Saccharomyces cerevisiae*

**Fig 5:**
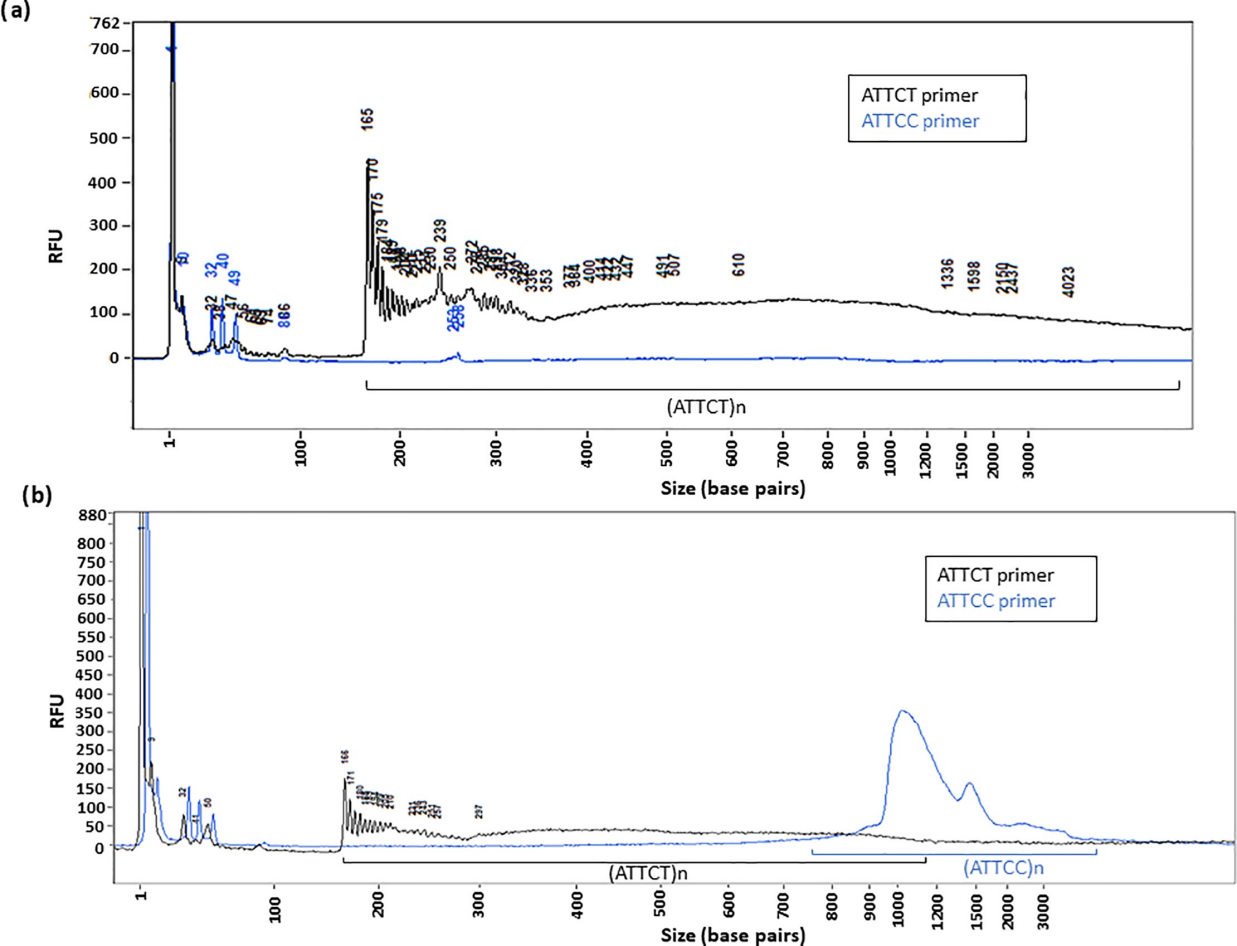
RP-FEMTO results of repeat maintenance upon transformation into a YAC in *Saccharomyces cerevisiae*. **(a)** RP-FEMTO results of genomic DNA using ATTCT (black line), ATTCC (blue line) internal primers of a pure ATTCT SCA10 pentanucleotide repeat transformed into a YAC and run on the RP-FEMTO using the small separation gel. As expected, no peak is present using the ATTCC primer since no ATTCC sequence should be present in this sample. **(b)** RP-FEMTO results of yeast genomic DNA using ATTCT (black line) or ATTCC (blue line) internal primers of an SCA10 pentanucleotide repeat containing an (ATTCC)_n_ interruption transformed into a YAC and run on the FEMTO Pulse system using the small separation gel. Using the RP-FEMTO methodology, we were able to determine a (ATTCT)_188_ and (ATTCC)_650_. There may exist some heterogeneity in the yeast population since there appears to be some overlap between the ATTCT repeat and ATTCC insertion.

To establish a yeast model of SCA10, large pentanucleotide repeat sequences derived from blood DNA from patients with SCA10 maintaining either a pure expanded (ATTCT)_n_ repeat or an (ATTCT)_n_ repeat with (ATTCC)_n_ interruption were transformed into a yeast artificial chromosome (YAC) in *Saccharomyces cerevisiae*. To determine whether the large repeat maintained its size and content in yeast we used the RP-FEMTO for high throughput sequence evaluation of these YACs. Fig 5 shows results from an RP-FEMTO run of two individual yeast integrants. A pure ATTCT pentanucleotide maintains a length of more than 4000 bp in the YAC (Fig 5A). Fig 5B shows a clone maintaining an (ATTCT)_n_ of about 1100 bp with (ATTCC)_n_ insertion of about 2700 bp occurring at about 650 bp of ATTCT. Since there appears to be an overlap between the (ATTCT)_n_ repeat and the (ATTCC)_n_ insertion, it is possible that there occurs some small amount of heterogeneity in the yeast population.

## Discussion

We demonstrated that the RP-FEMTO provides a fast and economical analysis of interrupting repeat sequence structures in large expanded repeats of SCA10. This technology may be applicable to other repeat expansions that are known to contain heterogeneous repeats at disease loci, including *ATXN8OS* in SCA8 [3], *BEAN* in SCA31 [4], *NOP56* in SCA36 [28], *DAB1* in SCA37 [6], *FXN* in Friedreich ataxia [29], *RFC1* in AR-CANVAS [17], *SAMD12*, *TNRC6A* and *RAPGEF2* in BAFME [13], *STARD7* in BAFME2 [14], *MARCHF6* in BAFME3 [15], *YEATS2* in BAFME4 [16], *DMPK* in DM1 [30], and *TCF4* in Fuchs endothelial corneal dystrophy (FECD) [31]. These internal heterogeneous repeats within the expanded allele may contribute to reduced penetrance and variable disease phenotypes. In large repeat expansions of fragile X syndrome [32] and C9ORF72-ALS/FTD [33] SMRT sequencing has shown no interrupting sequences although the number of expanded alleles studied was limited. In large repeat expansion diseases with other repeat insertions that may be several kilobases away from the flanking PCR primer, RP-Femto would be a good solution for detection of known sequence repeat insertions since only a very small amount of RP-PCR product would be necessary for detection as observed with the SCA10 patient samples. In these disorders, determination of the sequence composition of expanded repeat is important for diagnosing the disease, understanding the pathogenic mechanism, and developing therapeutic strategies. Large repeat expansions are currently difficult to sequence using typical sequencing methods. The size of the repeat as well as the potential secondary structures that the repeats can form can hinder the usual sequencing methods to examine the repeats. SMRT sequencing is one of the new technologies used today to be able to sequence such large repeat expansions. For SCA10, up to 7500 bp of pentanucleotide repeats were sequenced using SMRT sequencing. However, the limitation of SMRT sequencing is preferential sequencing of small repeat-size alleles, including normal alleles and small mutant alleles, and difficulties in generating circular consensus sequences of expanded SCA10 repeats containing large (ATCCC) repeat interruptions. We believe that this is due to the high GC content although the SMRT sequencing has been successfully applied to expanded GGGGCC repeats of C9ORF72-ALS/FTD [33] and CGG expansions in fragile X syndrome [32]. Secondary structures unique to ATCCC repeats may play a role in this hindrance to SMRT sequencing.

To bypass these obstacles, we used RP-FEMTO to determine pentanucleotide repeat sequence content and to determine repeat stability. Due to the intrinsic instability of repeat DNA in both patients and in laboratory systems, variable repeat lengths are possibly present within the same system or patient. SCA10 patients’ blood DNA showed great repeat length heterogeneity with the single molecule sequencing (SMRT sequencing). Examination of the same SCA10 patient samples with the RP-FEMTO showed that some cells may contain larger repeats than evident with SMRT sequencing and that there may be even more heterogeneity within the patient DNA sample. Due to the sensitivity of the FEMTO-Pulse system, even a small amount of PCR product will be detected, enabling amplification of very larger repeats that may be limited in the genomic DNA sample and may elude detection by other methods like Southern hybridization due to sensitivity or SMRT sequencing due to ratio of the rare repeat to the entire genomic DNA content. Southern hybridization would have the same drawback as SMRT sequencing in that with Southern hybridization it would be more likely to observe the majority repeat length (the repeat length that is highest in concentration) in a heterogenous DNA repeat population. Also in general, 5–10 ug of human genomic DNA is required for southern hybridization as opposed to the 50 pg requirement for RP-PCR.

The RP-FEMTO was essential for quickly assessing length and repeat motifs of pentanucleotide repeats after integration into a YAC present in the yeast *Saccharomyces cerevisiae*. The large repeat size and content of the repeat made it difficult to PCR and sequence using canonical PCR and sequencing methods. The RP-FEMTO overcomes this problem with only 25 ng of RP PCR product necessary for the analysis. The RP-FEMTO is an easy and quick methodology that allows for assessing many integrants simultaneously, albeit not individually at the single molecule level, for repeat length and repeat motifs in the yeast genome.

The RP-FEMTO can determine the configuration of known interrupting runs of heterogeneous repeat units in large repeat expansions but does not sequence expanded repeats. To use the RP-FEMTO effectively, users need to understand its limitations (Table 2). The RP-FEMTO cannot replace SMRT or Oxford Nanopore sequencing technologies but should be used to complement and enhance the utility of these sequencing technologies. We have not tested the Oxford Nanopore systems that can also generate long reads. With improving sequencing accuracy and efficiency of new sequencing technologies that can generate long reads of expanded repeats, the RP-FEMTO may become unnecessary in analysis of very large repeat expansions. However, until then, and even after then, this method should be useful for analysis of large repeat expansions in some disorders. We conclude that the RP-FEMTO is useful for a quick and cost-effective analysis of very large microsatellite repeat expansion, especially when the expanded repeat contains known runs of heterogeneous repeats.

**Table 2:** Advantages and disadvantages of RP-FEMTO analysis in comparison to SMRT sequencing.

Advantages	Disadvantages
Quick and economical analysis. The Genomic DNA 165 kb kit produces results in as little as 1.5 hours and up to 88 samples can be run simultaneously on one gel.	Only applicable when interrupting repeat units are known or can be predicted
Results can be obtained in one day. RP PCR is run directly on the FEMTO-Pulse machine	When individual repeat tracks vary in length, downstream peaks may become less defined
CG-rich repeat units are readily detectable	Non-repeat interruptions escape detections
Provides the approximate size of expanded allele	Unable to generate sequence reads
No bias for shorter alleles for detection	
Minimal amount of DNA only 50–500 pg of DNA required.	

## References

[1] Matsuura T, Yamagata T, Burgess DL, Rasmussen A, Grewal RP, Watase K, et al Large expansion of the ATTCT pentanucleotide repeat in spinocerebellar ataxia type 10. Nat Genet. 2000;26(2):191–4. 10.1038/79911 11017075

[2] McFarland KN, Liu J, Landrian I, Godiska R, Shanker S, Yu F, et al SMRT Sequencing of Long Tandem Nucleotide Repeats in SCA10 Reveals Unique Insight of Repeat Expansion Structure. PLoS One. 2015;10(8):e0135906 10.1371/journal.pone.0135906 26295943 PMC4546671

[3] Koob MD, Moseley ML, Schut LJ, Benzow KA, Bird TD, Day JW, et al An untranslated CTG expansion causes a novel form of spinocerebellar ataxia (SCA8). Nat Genet. 1999;21(4):379–84. 10.1038/7710 10192387

[4] Sato N, Amino T, Kobayashi K, Asakawa S, Ishiguro T, Tsunemi T, et al Spinocerebellar ataxia type 31 is associated with "inserted" penta-nucleotide repeats containing (TGGAA)n. Am J Hum Genet. 2009;85(5):544–57. 10.1016/j.ajhg.2009.09.019 19878914 PMC2775824

[5] Kobayashi H, Abe K, Matsuura T, Ikeda Y, Hitomi T, Akechi Y, et al Expansion of intronic GGCCTG hexanucleotide repeat in NOP56 causes SCA36, a type of spinocerebellar ataxia accompanied by motor neuron involvement. Am J Hum Genet. 2011;89(1):121–30. 10.1016/j.ajhg.2011.05.015 21683323 PMC3135815

[6] Seixas AI, Loureiro JR, Costa C, Ordonez-Ugalde A, Marcelino H, Oliveira CL, et al A Pentanucleotide ATTTC Repeat Insertion in the Non-coding Region of DAB1, Mapping to SCA37, Causes Spinocerebellar Ataxia. Am J Hum Genet. 2017;101(1):87–103. 10.1016/j.ajhg.2017.06.007 28686858 PMC5501871

[7] Brook JD, McCurrach ME, Harley HG, Buckler AJ, Church D, Aburatani H, et al Molecular basis of myotonic dystrophy: expansion of a trinucleotide (CTG) repeat at the 3' end of a transcript encoding a protein kinase family member. Cell. 1992;69(2):385 10.1016/0092-8674(92)90418-c 1568252

[8] Liquori CL, Ricker K, Moseley ML, Jacobsen JF, Kress W, Naylor SL, et al Myotonic dystrophy type 2 caused by a CCTG expansion in intron 1 of ZNF9. Science. 2001;293(5531):864–7. 10.1126/science.1062125 11486088

[9] Kremer EJ, Pritchard M, Lynch M, Yu S, Holman K, Baker E, et al Mapping of DNA instability at the fragile X to a trinucleotide repeat sequence p(CCG)n. Science. 1991;252(5013):1711–4. 10.1126/science.1675488 1675488

[10] Hagerman RJ, Leehey M, Heinrichs W, Tassone F, Wilson R, Hills J, et al Intention tremor, parkinsonism, and generalized brain atrophy in male carriers of fragile X. Neurology. 2001;57(1):127–30. 10.1212/wnl.57.1.127 11445641

[11] Dols-Icardo O, Garcia-Redondo A, Rojas-Garcia R, Sanchez-Valle R, Noguera A, Gomez-Tortosa E, et al Characterization of the repeat expansion size in C9orf72 in amyotrophic lateral sclerosis and frontotemporal dementia. Hum Mol Genet. 2014;23(3):749–54. 10.1093/hmg/ddt460 24057670

[12] Lodi R, Cooper JM, Bradley JL, Manners D, Styles P, Taylor DJ, et al Deficit of in vivo mitochondrial ATP production in patients with Friedreich ataxia. Proc Natl Acad Sci U S A. 1999;96(20):11492–5. 10.1073/pnas.96.20.11492 10500204 PMC18061

[13] Ishiura H, Doi K, Mitsui J, Yoshimura J, Matsukawa MK, Fujiyama A, et al Expansions of intronic TTTCA and TTTTA repeats in benign adult familial myoclonic epilepsy. Nat Genet. 2018;50(4):581–90. 10.1038/s41588-018-0067-2 29507423

[14] Corbett MA, Kroes T, Veneziano L, Bennett MF, Florian R, Schneider AL, et al Intronic ATTTC repeat expansions in STARD7 in familial adult myoclonic epilepsy linked to chromosome 2. Nat Commun. 2019;10(1):4920 10.1038/s41467-019-12671-y 31664034 PMC6820779

[15] Florian RT, Kraft F, Leitao E, Kaya S, Klebe S, Magnin E, et al Unstable TTTTA/TTTCA expansions in MARCH6 are associated with Familial Adult Myoclonic Epilepsy type 3. Nat Commun. 2019;10(1):4919 10.1038/s41467-019-12763-9 31664039 PMC6820781

[16] Yeetong P, Pongpanich M, Srichomthong C, Assawapitaksakul A, Shotelersuk V, Tantirukdham N, et al TTTCA repeat insertions in an intron of YEATS2 in benign adult familial myoclonic epilepsy type 4. Brain. 2019;142(11):3360–6. 10.1093/brain/awz267 31539032

[17] Cortese A, Simone R, Sullivan R, Vandrovcova J, Tariq H, Yau WY, et al Author Correction: Biallelic expansion of an intronic repeat in RFC1 is a common cause of late-onset ataxia. Nat Genet. 2019;51(5):920. 10.1038/s41588-019-0422-y PMC6730635 31028356

[18] Mootha VV, Hussain I, Cunnusamy K, Graham E, Gong X, Neelam S, et al TCF4 Triplet Repeat Expansion and Nuclear RNA Foci in Fuchs' Endothelial Corneal Dystrophy. Invest Ophthalmol Vis Sci. 2015;56(3):2003–11. 10.1167/iovs.14-16222 25722209 PMC4373545

[19] Matsuura T, Fang P, Pearson CE, Jayakar P, Ashizawa T, Roa BB, et al Interruptions in the expanded ATTCT repeat of spinocerebellar ataxia type 10: repeat purity as a disease modifier? Am J Hum Genet. 2006;78(1):125–9. 10.1086/498654 16385455 PMC1380209

[20] McFarland KN, Liu J, Landrian I, Zeng D, Raskin S, Moscovich M, et al Repeat interruptions in spinocerebellar ataxia type 10 expansions are strongly associated with epileptic seizures. Neurogenetics. 2014;15(1):59–64. 10.1007/s10048-013-0385-6 24318420 PMC4038098

[21] Loureiro JR, Oliveira CL, Sequeiros J, Silveira I. A repeat-primed PCR assay for pentanucleotide repeat alleles in spinocerebellar ataxia type 37. J Hum Genet. 2018;63(9):981–7. 10.1038/s10038-018-0474-3 29891931

[22] Musova Z, Mazanec R, Krepelova A, Ehler E, Vales J, Jaklova R, et al Highly unstable sequence interruptions of the CTG repeat in the myotonic dystrophy gene. Am J Med Genet A. 2009;149A(7):1365–74. 10.1002/ajmg.a.32987 19514047

[23] Moseley ML, Schut LJ, Bird TD, Koob MD, Day JW, Ranum LP. SCA8 CTG repeat: en masse contractions in sperm and intergenerational sequence changes may play a role in reduced penetrance. Hum Mol Genet. 2000;9(14):2125–30. 10.1093/hmg/9.14.2125 10958651

[24] Mizuguchi T, Toyota T, Adachi H, Miyake N, Matsumoto N, Miyatake S. Detecting a long insertion variant in SAMD12 by SMRT sequencing: implications of long-read whole-genome sequencing for repeat expansion diseases. J Hum Genet. 2019;64(3):191–7. 10.1038/s10038-018-0551-7 30559482

[25] Matsuura T, Ashizawa T. Polymerase chain reaction amplification of expanded ATTCT repeat in spinocerebellar ataxia type 10. Ann Neurol. 2002;51(2):271–2. 10.1002/ana.10049 11835387

[26] McFarland KN, Liu J, Landrian I, Gao R, Sarkar PS, Raskin S, et al Paradoxical effects of repeat interruptions on spinocerebellar ataxia type 10 expansions and repeat instability. Eur J Hum Genet. 2013;21(11):1272–6. 10.1038/ejhg.2013.32 23443018 PMC3798839

[27] Schule B, McFarland KN, Lee K, Tsai YC, Nguyen KD, Sun C, et al Parkinson's disease associated with pure ATXN10 repeat expansion. NPJ Parkinsons Dis. 2017;3:27 10.1038/s41531-017-0029-x 28890930 PMC5585403

[28] Obayashi M, Stevanin G, Synofzik M, Monin ML, Duyckaerts C, Sato N, et al Spinocerebellar ataxia type 36 exists in diverse populations and can be caused by a short hexanucleotide GGCCTG repeat expansion. J Neurol Neurosurg Psychiatry. 2015;86(9):986–95. 10.1136/jnnp-2014-309153 25476002

[29] Ohshima K, Sakamoto N, Labuda M, Poirier J, Moseley ML, Montermini L, et al A nonpathogenic GAAGGA repeat in the Friedreich gene: implications for pathogenesis. Neurology. 1999;53(8):1854–7. 10.1212/wnl.53.8.1854 10563639

[30] Cumming SA, Hamilton MJ, Robb Y, Gregory H, McWilliam C, Cooper A, et al De novo repeat interruptions are associated with reduced somatic instability and mild or absent clinical features in myotonic dystrophy type 1. Eur J Hum Genet. 2018;26(11):1635–47. 10.1038/s41431-018-0156-9 29967337 PMC6189127

[31] Hafford-Tear NJ, Tsai YC, Sadan AN, Sanchez-Pintado B, Zarouchlioti C, Maher GJ, et al CRISPR/Cas9-targeted enrichment and long-read sequencing of the Fuchs endothelial corneal dystrophy-associated TCF4 triplet repeat. Genet Med. 2019. 10.1038/s41436-019-0453-x PMC6752322 30733599

[32] Loomis EW, Eid JS, Peluso P, Yin J, Hickey L, Rank D, et al Sequencing the unsequenceable: expanded CGG-repeat alleles of the fragile X gene. Genome Res. 2013;23(1):121–8. 10.1101/gr.141705.112 23064752 PMC3530672

[33] Ebbert MTW, Farrugia SL, Sens JP, Jansen-West K, Gendron TF, Prudencio M, et al Long-read sequencing across the C9orf72 'GGGGCC' repeat expansion: implications for clinical use and genetic discovery efforts in human disease. Mol Neurodegener. 2018;13(1):46 10.1186/s13024-018-0274-4 30126445 PMC6102925

