# Correction: Pulse-Field capillary electrophoresis of repeat-primed PCR amplicons for analysis of large repeats in Spinocerebellar Ataxia Type 10

**DOI:** 10.1371/journal.pone.0231746

**Published:** 2020-04-16

**Authors:** Vera Hashem, Anjana Tiwari, Brittani Bewick, Helio A. G. Teive, Mariana Moscovich, Birgitt Schüle, Khalaf Bushara, Matt Bower, Astrid Rasmussen, Yu-Chih Tsai, Tyson Clark, Karen McFarland, Tetsuo Ashizawa

The sixth author's name is spelled incorrectly. The correct name is: Birgitt Schüle. The correct citation is: Hashem V, Tiwari A, Bewick B, Teive HAG, Moscovich M, Schüle B, et al. (2020) Pulse-Field capillary electrophoresis of repeat-primed PCR amplicons for analysis of large repeats in Spinocerebellar Ataxia Type 10. PLoS ONE 15(3): e0228789. https://doi.org/10.1371/journal.pone.0228789
